# Age- and Sex-Specific Plasticity in Dopamine Transporter Function Revealed by Food Restriction and Exercise in a Rat Activity-Based Anorexia Paradigm[Fn FN3]

**DOI:** 10.1124/jpet.119.260794

**Published:** 2019-11

**Authors:** T. Lee Gilman, W. Anthony Owens, Christina M. George, Lauren Metzel, Melissa Vitela, Livia Ferreira, Melodi A. Bowman, Georgianna G. Gould, Glenn M. Toney, Lynette C. Daws

**Affiliations:** Department of Cellular and Integrative Physiology (T.L.G., W.A.O., C.M.G., L.M., M.V., L.F., M.A.B., G.G.G., G.M.T., L.C.D.), Addiction Research, Treatment & Training Center of Excellence (T.L.G., L.C.D.), Center for Biomedical Neuroscience (G.M.T., L.C.D.), and Department of Pharmacology (L.C.D.), University of Texas Health Science Center at San Antonio, San Antonio, Texas

## Abstract

Eating disorders such as anorexia typically emerge during adolescence, are characterized by engagement in compulsive and detrimental behaviors, and are often comorbid with neuropsychiatric disorders and drug abuse. No effective treatments exist. Moreover, anorexia lacks adolescent animal models, contributing to a poor understanding of underlying age-specific neurophysiological disruptions. To evaluate the contribution of dopaminergic signaling to the emergence of anorexia-related behaviors during the vulnerable adolescent period, we applied an established adult activity-based anorexia (ABA) paradigm (food restriction plus unlimited exercise access for 4 to 5 days) to adult and adolescent rats of both sexes. At the end of the paradigm, measures of plasma volume, blood hormone levels, dopamine transporter (DAT) expression and function, acute cocaine-induced locomotion, and brain water weight were taken. Adolescents were dramatically more affected by the ABA paradigm than adults in all measures. In vivo chronoamperometry and cocaine locomotor responses revealed sex-specific changes in adolescent DAT function after ABA that were independent of DAT expression differences. Hematocrit, insulin, ghrelin, and corticosterone levels did not resemble shifts typically observed in patients with anorexia, though decreases in leptin levels aligned with human reports. These findings are the first to suggest that food restriction in conjunction with excessive exercise sex-dependently and age-specifically modulate DAT functional plasticity during adolescence. The adolescent vulnerability to this relatively short manipulation, combined with blood measures, evidence need for an optimized age-appropriate ABA paradigm with greater face and predictive validity for the study of the pathophysiology and treatment of anorexia.

**SIGNIFICANCE STATEMENT:**

Adolescent rats exhibit a distinctive, sex-specific plasticity in dopamine transporter function and cocaine response after food restriction and exercise access; this plasticity is both absent in adults and not attributable to changes in dopamine transporter expression levels. These novel findings may help explain sex differences in vulnerability to eating disorders and drug abuse during adolescence.

## Introduction

Eating disorders afflict ≥3% of individuals in developed countries, and disproportionately impact females over males by more than double (Smink et al., 2014; Keski-Rahkonen and Mustelin, 2016). These disorders, which include anorexia nervosa (AN), bulimia nervosa, and binge-eating disorder, most often emerge during adolescence (Herpertz-Dahlmann, 2015). Indeed, adolescence is characterized not only by an increased risk of eating disorders, but also other neuropsychiatric disorders, as well as drug abuse and similar risky or compulsive behaviors (Andersen, 2003; Chambers et al., 2003; Paus et al., 2008; Arain et al., 2013; Holder and Blaustein, 2014).

Adolescence coincides with a particularly sensitive maturation period for the dopaminergic system (Chambers et al., 2003; Casey et al., 2008; Ernst et al., 2009; Novick et al., 2011; Sinclair et al., 2014; Fuhrmann et al., 2015; Niwa et al., 2016). Dopamine (DA) is a catecholamine neurotransmitter important for modulation of eating (Rolls et al., 1974; Yoshida et al., 1992; Volkow et al., 2003; Davis et al., 2009), motor activity (Andén et al., 1973; Baldo et al., 2002; Gallardo et al., 2014; Robertson et al., 2015; Felger and Treadway, 2017), emotion (Herman et al., 1982; Salgado-Pineda et al., 2005; Kienast et al., 2008; Belujon and Grace, 2015), impulsivity (van Gaalen et al., 2006; Buckholtz et al., 2010; Pine et al., 2010; Dalley and Robbins, 2017), and reward (Nestler and Carlezon, 2006; Johnson and Kenny, 2010; Eshel et al., 2016). Eating disorders, particularly AN and bulimia nervosa, are characterized by drastic changes in eating and/or activity behaviors that, when sufficiently rewarding to the individual’s desired outcome (e.g., dramatic weight loss), can lead to compulsive engagement in such behavior despite severe deterioration in health or lifestyle. Consequently, DA pathophysiology is suspected to underlie, at least in part, adolescent-onset eating disorders (Barry and Klawans, 1976; Golden and Shenker, 1994; Frank et al., 2005; Frieling et al., 2010; Scherag et al., 2010; Kaye et al., 2013).

AN consistently has the highest mortality rate of all eating disorders (Arcelus et al., 2011; Preti et al., 2011; Keshaviah et al., 2014). Yet effective pharmacological treatments do not exist for *any* eating disorders. Antidepressant drugs, though beneficial for frequently comorbid mood disturbances, appear otherwise ineffective in treating AN, and antipsychotic drugs have marginal if any effectiveness [see reviews by Carrera et al. (2014), Zipfel et al. (2015)]. One potential explanation for such weak treatment effectiveness is a lack of AN animal models. As with most neuropsychiatric disorders, modeling pathophysiology in animals such as rodents is inherently constrained by species differences and questions of emotional and intellectual complexity [refer to Gutierrez (2013), Bale et al. (2019)]. Nonetheless, introduction of specific disorder components, such as the varied physical and environmental stressors in the chronic mild stress model of depression (Willner, 2016), can be advantageous both for understanding neurophysiological disruptions (face validity) and for screening potential therapeutics (predictive validity). Here, we explored use of an adult activity-based anorexia (ABA) paradigm in early adolescence (postnatal day 30, P30) to examine how dopaminergic disruptions from food restriction and/or exercise might differ as a function of sex in adolescents and adults. Until now, animal investigations into anorexia have predominantly used adults, with the few examining adolescent animals missing the vulnerable *early* adolescent period (Kinzig and Hargrave, 2010; Aoki et al., 2012; Barbarich-Marsteller et al., 2013a,b; Chowdhury et al., 2014; Frintrop et al., 2018a,b) during which these behaviors typically emerge in humans (approx. age 13 years) (Micali et al., 2014; Nagl et al., 2016).

The ABA paradigm is based upon work in the 1950s and 1960s that observed significantly enhanced running wheel activity in adult male rats under conditions of food restriction (Hall et al., 1953), to the extent that after approximately 2 weeks, animals ran themselves to death rather than eat when food was available (Routtenberg and Kuznesof, 1967). Current paradigm iterations closely resemble these original findings, involving unlimited running wheel access (exercise) plus duration-based (rather than quantity-based) food restriction (1 hour/day) (Carrera et al., 2014). To assess dopaminergic disruptions in the ABA paradigm, we measured DA transporter (DAT) function after food restriction, exercise, or their combination (ABA) in adult and adolescent rats of both sexes. Because DA uptake by DAT is a primary regulatory mechanism of dopamine signaling duration, measuring DAT function provides an excellent indication of dopaminergic tone (Cass and Gerhardt, 1994; Zahniser et al., 1999; Daws et al., 2002; Sabeti et al., 2003). To our knowledge, this is the first assessment of DAT function in an ABA paradigm.

## Materials and Methods

### 

#### Animals.

Sprague-Dawley rats were bred in-house from breeders purchased through Taconic (NTac:SD; Rensselaer, NY), and all animals were maintained at 24°C on a 12:12 light/dark cycle, lights on at 0400 hours. Rats were weaned at P21, with P0 as day of birth. Rats were group-housed, two to three per cage, with same-sex littermates on 7090 Teklad sani-chip bedding (Envigo, East Millstone, NJ) and provided ad libitum access to water and Teklad LM-485 mouse/rat sterilizable diet 7012 chow (Envigo) until commencement of experimental manipulations. All experiments were approved by the University of Texas Health Science Center at San Antonio Institutional Animal Care and Use Committee, and complied with the National Research Council’s *Guide for the Care and Use of Laboratory Animals*, 8th Ed.

#### Activity-Based Anorexia Paradigm.

**Fig. 1.**
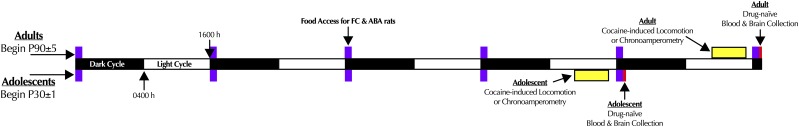
Timeline of experiments. Adult male and female rats were singly housed in cages directly adjoining running wheels at postnatal day 90 (P90, ±5 days), whereas adolescent male and female rats were housed in the same setup at P30 (±1 day). Cage control (CC) and exercise control (EC) animals had 24-hour access to food, whereas food restricted control (FC) and activity-based anorexia (ABA) animals were given access to food for 1 hour per day (purple blocks) at the onset of the dark cycle (1600 hours, 12:12 dark/light cycle, lights on at 0400 hours). Adolescents went through the paradigm for 4 days, adults for 5 days. At the end of the paradigm, animals underwent one of three endpoints: chronoamperometry (between 0900 and 1500 hours; yellow block), cocaine-induced locomotion (between 1000 and 1500 hours; yellow block), or postprandial drug-naive blood and brain collection (1700 hours, red block).

Adolescent (P30 ± 1 day) and adult (P90 ± 5 days) male and female rats were randomly assigned to one of four treatment conditions: cage control (CC), exercise control (EC), food restricted control (FC), and ABA. Initial body weights were not different across treatment conditions within any age group (see Supplemental Table S1). Body weights, and food and water consumed, were measured once daily between 1500 and 1600 hours. Animals always began in the paradigm at 1600 hours; adults continued for 5 days [a standard adult paradigm lasts for between 8 and 14 days (Routtenberg and Kuznesof, 1967; Carrera et al., 2014)], but adolescents were restricted to 4 days due to survival issues during pilot chronoamperometry and cocaine-induced locomotion endpoints at 5 days. On the final day of their respective paradigm, rats were assigned to one of three endpoints (Fig. 1): blood and brain collection from drug-naive rats (i.e., not previously used in chronoamperometry or locomotor experiments) for blood hormone and quantitative autoradiography analyses; locomotor assay to measure acute, dose-dependent effects of cocaine, a DAT blocker; or in vivo high-speed chronoamperometry for measurement of DA uptake in the DAT-rich dorsal striatum, a brain region important for feeding behavior (Palmiter, 2008). Additional details are in the Supplemental Material.

#### Chemicals and Reagents.

All chemicals and reagents were purchased from Sigma Aldrich (St. Louis, MO) unless otherwise indicated.

#### Locomotor Assay.

Testing occurred between 1000 and 1500 hours on the final paradigm day. Locomotor activity was measured using beam breaks quantified in 1-minute bins with Multi-Varimex software (v2.10; Columbus Instruments, Columbus, OH). Animals first underwent a 45-minute habituation session; thereafter, rats received injections of vehicle (0.9% NaCl, saline; 1 ml/kg, i.p.), then cocaine at increasing doses (3.2, 5.6, and 10 mg/kg) such that cumulative doses were 3.2, 8.8, and 18.8 mg/kg, with locomotor activity recorded for 15 minutes following each injection. For statistical comparisons, area under the curve (AUC) was calculated for the 15 minute period after saline and each cocaine injection using GraphPad Prism (v7.0e; GraphPad Software, La Jolla, CA). Additional details are in the Supplemental Material.

#### In Vivo High-Speed Chronoamperometry.

Chronoamperometry involves measurement of the clearance of increasing amounts of exogenously applied DA from the extracellular space, at a time resolution of 100 milliseconds and spatial resolution of micrometers. This technique is an established tool for measuring DAT function in vivo and was performed as previously described (Baladi *et al.*, 2015; Daws *et al.*, 2016). Additional details are in the Supplemental Material.

#### [^125^I] RTI-55 Quantitative Autoradiography.

Autoradiography was performed as previously detailed (Coulter et al., 1996). Additional details are in the Supplemental Material.

#### Statistical Analyses.

Data were analyzed in each age group with a two-way (sex × condition) analysis of variance (ANOVA) in NCSS (v12.0.9; NCSS, LLC, Kaysville, UT) with Tukey-Kramer’s post-hoc tests where appropriate to a priori compare across sex within condition, or across condition within sex. Statistical outcomes of three-way (day × sex × condition) repeated-measures ANOVA analyses of weights, with Geisser-Greenhouse correction for within-subjects analyses, are provided in Supplemental Table S2 for the drug-naive cohort of animals. Three-way (pmol of DA × sex × condition) repeated-measures ANOVA analyses of DA clearance rate, with Geisser-Greenhouse correction for within-subjects analyses, are provided in Supplemental Table S8. Data were graphed using GraphPad Prism (v7.0e; GraphPad Software). Significance was set a priori at *P* < 0.05. Additional details are in the Supplemental Material.

## Results

### Body Weights

**Fig. 2.**
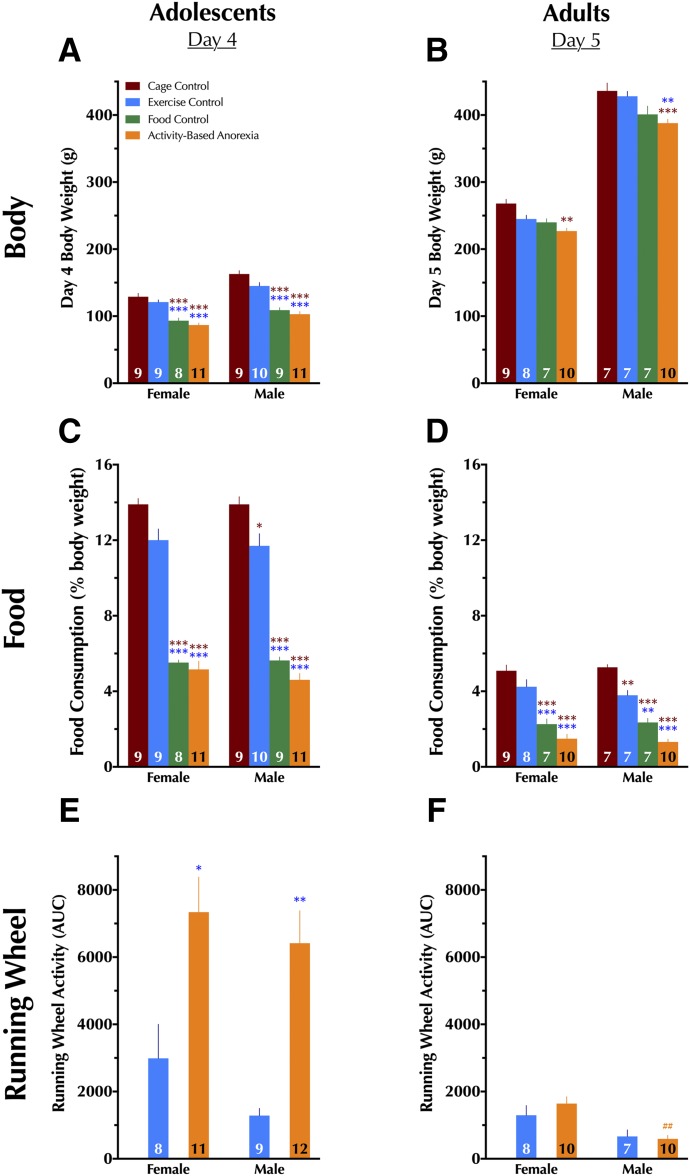
Final day body weight, food consumption, and running wheel measures in drug-naive rats. The final day in the experimental paradigm for adolescents was day 4, whereas the final day for adults was day 5. Because animals used for chronoamperometry and cocaine-induced locomotion were removed prior to completion of their full fourth (adolescents) or fifth (adults) day, only data from drug-naive rats are shown here. (A) Adolescent day 4 body weight; (B) adult day 5 body weight; (C) adolescent and (D) adult food consumption over the past 23 hours as a percentage of the individual animal’s body weight; (E) adolescent and (F) adult running wheel activity over the last 23 hours for EC and ABA animals. No running wheel activity is shown for CC and FC animals because their wheels were locked and therefore they could not run in the wheel. Data were analyzed within each age group with a two-way (sex × condition) ANOVA with Tukey-Kramer’s post-hoc tests where appropriate. Data are graphed as mean + S.E.M. *P < 0.05; **P < 0.01; ***P < 0.001 vs. same-sex condition, indicated by color of asterisk(s), within the same age group. ^##^*P* < 0.01 vs. same condition in females of same age group. Number of animals is indicated at base of each bar.

Initial weights were not different across treatment conditions in the drug-naive cohort, though expected sex differences in initial (day 0) weights were observed (Supplemental Table S1). By the end of the 4 or 5 day paradigm for adolescents and adults, respectively, significant weight differences across treatment conditions were noted. Day-by-day graphs of the time courses of body weights are provided in Supplemental Fig. S1, A–D, and three-way repeated-measure ANOVA outcomes relevant to these day-by-day measures are reported in Supplemental Table S2. Because all blood hormone, acute cocaine-induced locomotion, chronoamperometry, and autoradiography outcome measures took place on the final day of the paradigm, we focus here on final day weights, shown in Fig. 2.

#### Adolescents.

As with day 0 weights, a significant main effect of sex was observed for day 4 body weights [F(1,68) = 55.7, *P* < 0.001), but unlike day 0 weights, a significant main effect of treatment condition was also detected [F(3,68) = 69.3, *P* < 0.001]. No interactions between treatment and sex were detected. FC and ABA day 4 body weights were lower than both CC and EC groups within the same sex.

#### Adults.

Though no significant sex × condition interaction was observed, significant main effects of sex (F(1,57) = 1039, *P* < 0.001) and condition (F(3,57) = 15.0, *P* < 0.001) were found for day 5 body weights. Within adult females, only ABA animals had day 5 body weights different from CC animals. Weights of adult male ABA animals at day 5 were less than both CC and EC males.

### Food Consumption as Percent Body Weight

Given the significant differences in body weights at the end of the paradigms for adolescent and adult rats in the drug-naive cohort, food consumption for each 24 hour period of the paradigm was calculated for each animal as a percentage of their previous day’s body weight. Time-course graphs of these measures are presented in Supplemental Fig. S1, E–H, and as with body weights, we focus here on the final day of the adolescent (day 4) and adult (day 5) paradigms. Three-way repeated-measure ANOVA outcomes are reported in Supplemental Table S2.

#### Adolescents.

A significant main effect of condition [(F3,68) = 222, *P* < 0.001] was observed, with FC and ABA adolescents consuming less food than their EC and CC counterparts. No significant interaction between condition and sex, and no significant main effect of sex, were found. Adolescent EC males also consumed less food based on their body weight than CCs (Fig. 2C).

#### Adults.

As with adolescents, condition displayed a significant main effect [F(3,57) = 92.5, *P* < 0.001]. No significant main effect of sex was indicated, nor was a significant condition × sex interaction. Food intake was reduced in female FC and ABA conditions compared with female EC and CC conditions. Adult male body weight–based food intake on day 5 was reduced in EC, FC, and ABA animals relative to CC males. Male FC and ABA rats further exhibited reduced food intake relative to male EC animals (Fig. 2D).

### Running Wheel Activity

**Fig. 3.**
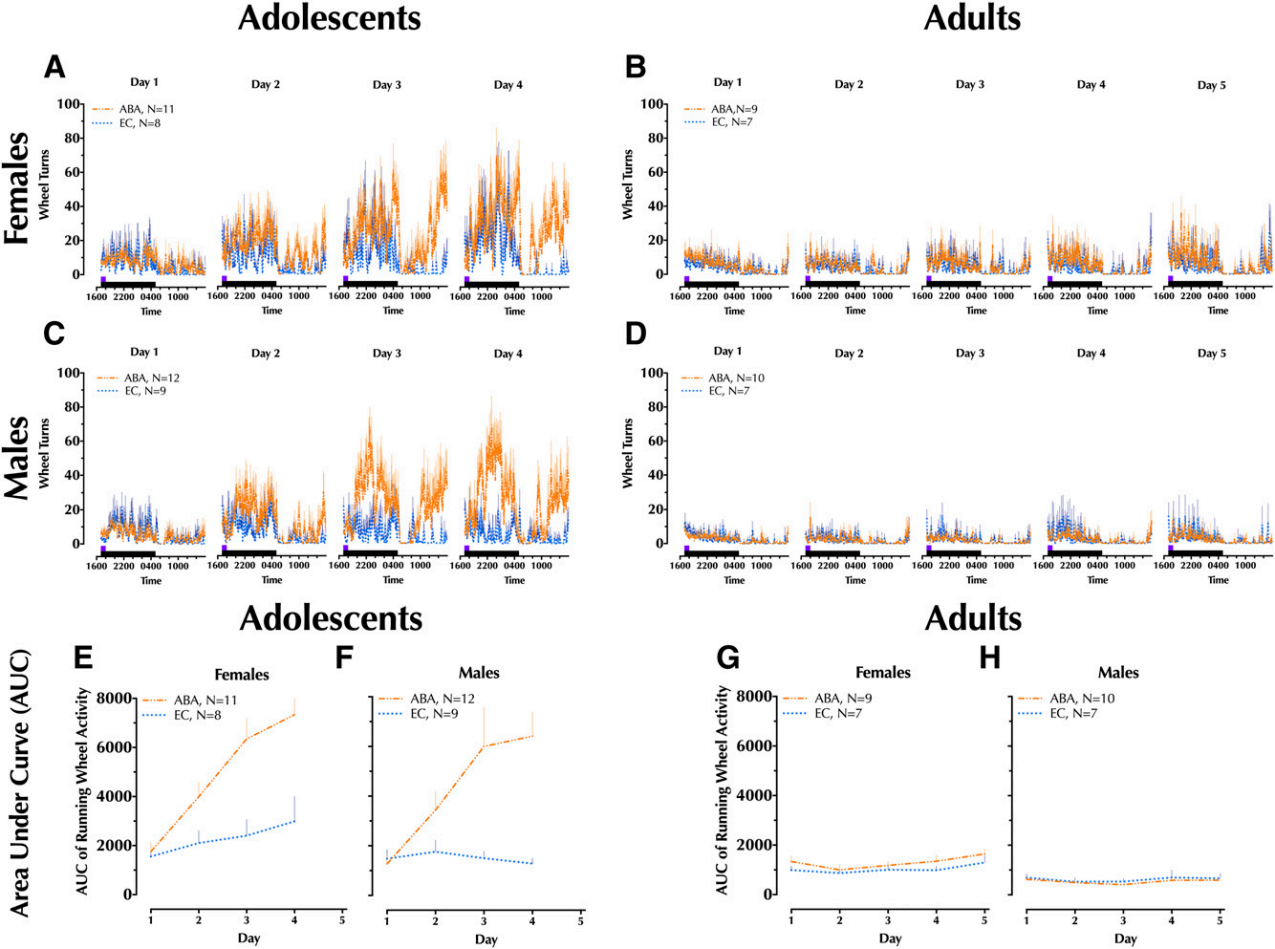
Daily running wheel measures in drug-naive rats. Adolescents were run in the experimental paradigm for 4 days, whereas adults were run for 5 days. Because animals used for chronoamperometry and cocaine-induced locomotion were removed prior to completion of their full fourth (adolescents) or fifth (adults) day, only data from drug-naive rats are shown here. Collected in 5 minute bins, 23 hours of running wheel activity were recorded each day for (A) adolescent female and (B) male running wheel activity over four paradigm days; (C) adult female and (D) male running wheel activity over five paradigm days. Area under the curve (AUC) was calculated for each day from the graphs in (A–D), and are shown for (E) adolescent female and (F) male animals; (G) adult female and (H) male animals. No running wheel activity is shown for CC and FC animals, because their wheels were locked and therefore they could not run in the wheel. Only the final day of wheel running activity was analyzed (refer to Fig. 2, E and F). Data are graphed as mean + S.E.M. Number of animals is indicated in line legend: exercise control (EC), activity-based anorexia (ABA).

Only animals in the EC and ABA conditions had unlocked running wheels (Fig. 3, A–D), so these two conditions were analyzed within age and across sex and condition. Area under the curve (AUC) was calculated for 23 hours during each paradigm day, and these AUC data per day are shown in Fig. 3, E–H, with corresponding three-way repeated-measure ANOVA outcomes reported in Supplemental Table S3. Final paradigm day 4 AUCs for adolescents are in Fig. 2E, and final day 5 for adults are in Fig. 2F.

#### Adolescents.

A significant main effect of condition was detected [F(1,36) = 25.9, *P* < 0.001], with both female and male ABA animals exhibiting greater day 4 running wheel activity relative to their same-sex EC counterparts (Fig. 2E). Sex × condition interaction did not reach significance, nor did a main effect of sex.

#### Adults.

There was no significant main effect of condition in adults, nor was there a significant sex × condition interaction. However, a main effect of sex was significant [F(1,31) = 17.1, *P* < 0.001], with this driven by greater running wheel activity in female ABA animals compared with male ABA (Fig. 2F).

### Blood Hormone and Other Physiologic Measures

See Supplemental Results and Supplemental Tables S4 and S5 for full details. Briefly, relative to same-sex CCs, circulating leptin and corticosterone were reduced in adolescent female ABA and FC groups, and adolescent female FCs also exhibited reduced ghrelin and increased insulin postprandial. Adolescent male blood hormone changes were similar for leptin and insulin, though no ghrelin or corticosterone differences relative to same-sex CCs were detected. In adults, only leptin levels were reduced compared to CCs in ABA animals (both sexes) and FCs (males). Brain water weights and plasma osmolality were unaffected in all groups. Hematocrit was elevated in adolescent ABAs (both sexes) compared to CCs, whereas only plasma protein was reduced selectively in adolescent female ABAs. No changes in either hematocrit or plasma protein were detected in any adults.

### Locomotor Activity

AUC for locomotor activity, measured by minute-to-minute beam breaks, was calculated for the 15 minutes immediately following a saline injection, and for the combined three 15 minute periods immediately following three successive individual doses of cocaine (3.2, 5.6, 10 mg/kg). These AUCs were then analyzed to compare locomotor responses after injections.

#### Locomotor Activity after Saline.

**Fig. 4.**
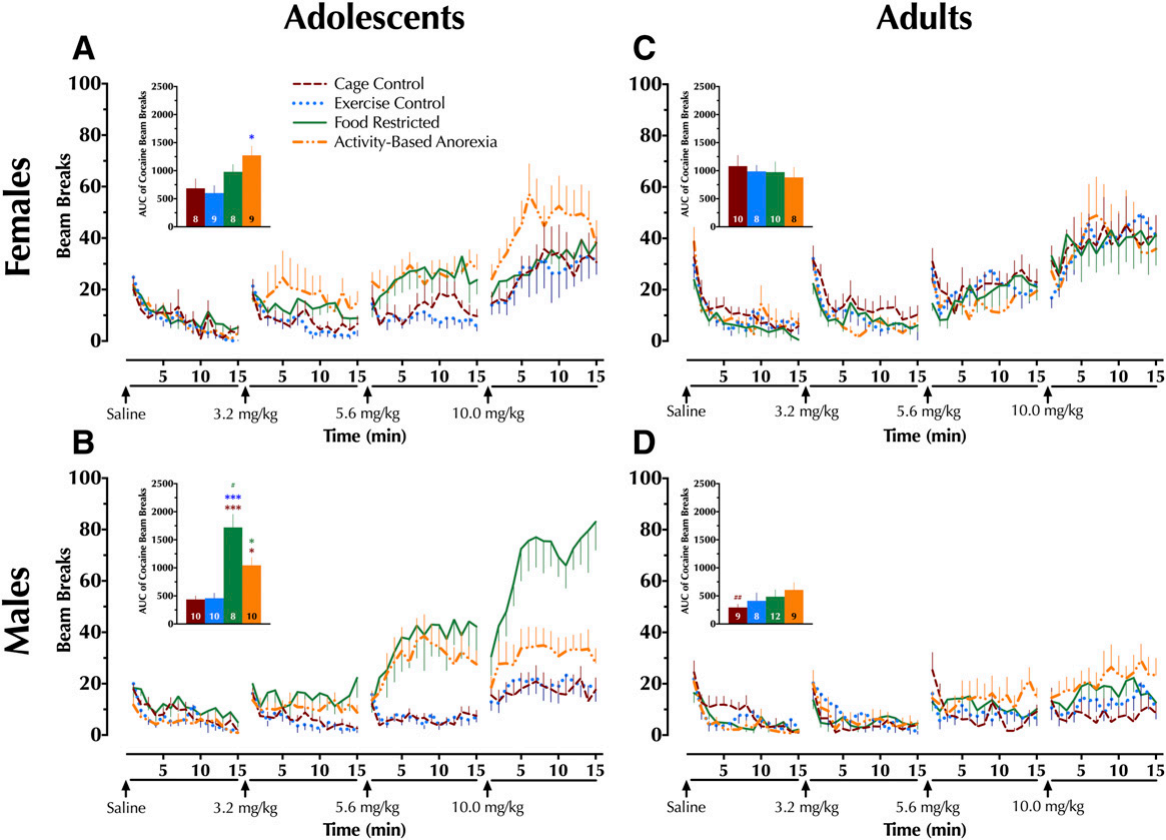
Cocaine-induced locomotion. Rats were habituated to activity chambers for 45 minutes, then injected first with saline (1 ml/kg), then increasing individual doses of cocaine (3.2, 5.6, and 10 mg/kg) at 15 minute intervals in a cumulative-dosing paradigm. Activity was quantified as the number of beam breaks in 1 minute bins (lines in graphs), and the area under the curve (AUC) for all three cocaine doses is cumulatively shown in inset bar graphs for adolescent (A) female and (B) male and adult (C) female and (D) male rats. AUC data (inset bar graphs) were analyzed within each age group with a two-way (sex × condition) ANOVA with Tukey-Kramer’s post-hoc tests where appropriate. Data are graphed as mean ± S.E.M. *P < 0.05; ***P < 0.001 vs. same-sex condition, indicated by color of asterisk(s), within the same age group. ^#^*P* < 0.05; ^##^*P* < 0.01 vs. same condition in females of same age group. Number of animals is indicated at base of each bar.

Saline AUCs are described in Supplemental Table S6, with corresponding time courses for saline and cocaine graphed in Fig. 4. For saline AUCs, no significant differences across treatment conditions were observed in either age group (Supplemental Table S6).

#### Locomotor Activity after Cocaine.

Cocaine AUCs are inclusive of all three individual doses (3.2, 5.6, 10 mg/kg).

##### Adolescents

A significant sex × condition interaction was observed in the cocaine AUCs of adolescent rats [F(3,64) = 5.11, *P* < 0.01]. Adolescent female ABA animals exhibited greater cocaine AUCs than EC counterparts (Fig. 4A). In contrast to adolescent females, adolescent males in the FC condition displayed greater cocaine AUCs than all other male conditions, and compared to female FC counterparts (Fig. 4B). Adolescent male ABA animals also exhibited higher cocaine-induced locomotion than male CC rats.

##### Adults

A main effect of sex was detected [F(1,66) = 25.4, *P* < 0.001], with female CC rats displaying a higher cocaine AUC than male CCs (Fig. 4, C and D). Condition did not show a significant main effect, nor was there a significant sex × condition interaction.

### In Vivo High-Speed Chronoamperometry

For chronoamperometry, signal amplitudes at the highest amount of DA infused (100 pmol) were first evaluated to confirm that these were not significantly affected by treatment condition (Supplemental Table S7). The clearance rate of DA from the extracellular space was then plotted against picomoles of DA infused.

#### Adolescents.

**Fig. 5.**
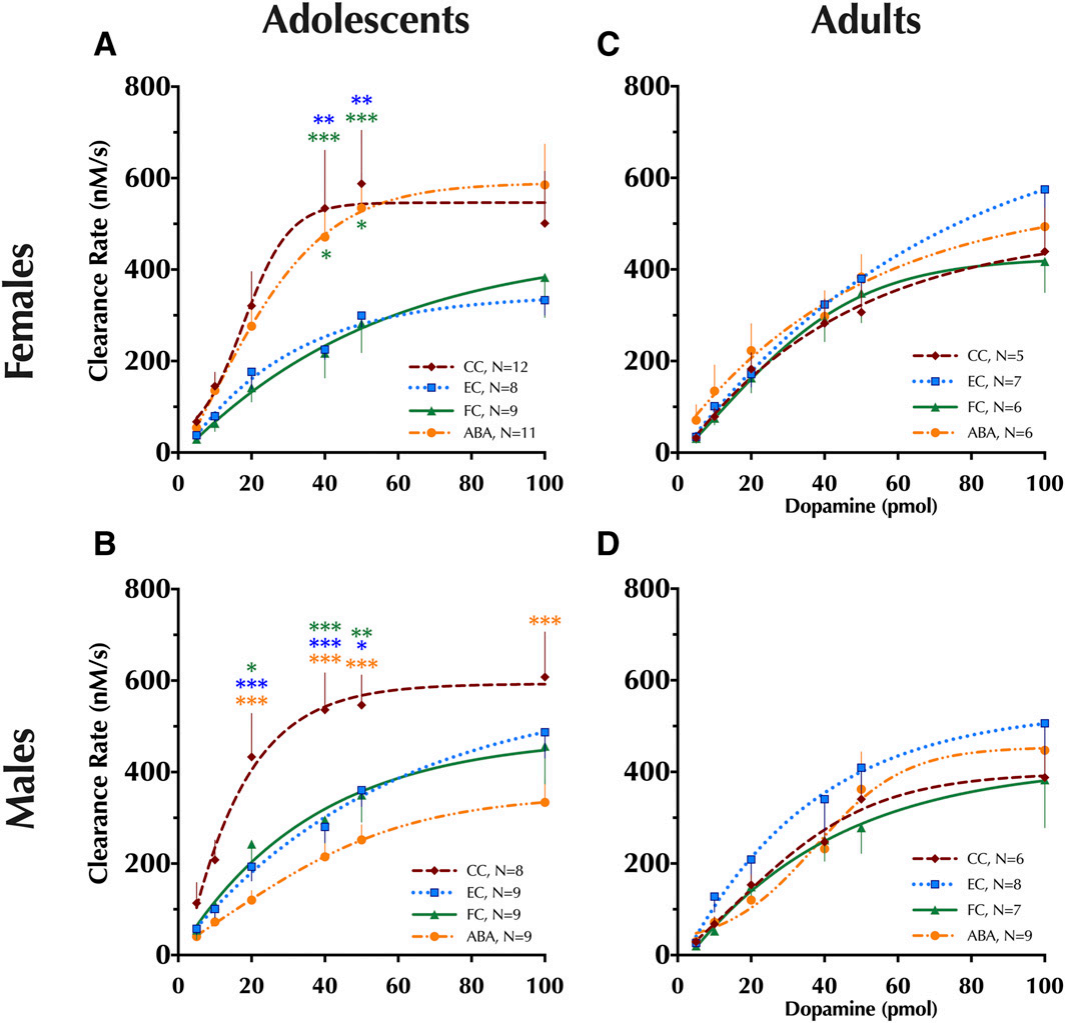
Striatal dopamine clearance measured using in vivo high-speed chronoamperometry. Clearance rate of dopamine in the striatum of adolescent (A) female and (B) male rats and adult (C) female and (D) male rats is shown for increasing amounts of exogenously infused dopamine. Four-parameter logistic regressions with baseline constrained at 0 were used to generate curve fits. Data analyzed within each age and sex group using a repeated-measures (pmol DA × condition) ANOVA with Tukey-Kramer’s post-hoc tests where appropriate. Data are graphed as mean ± S.E.M. *P < 0.05; **P < 0.01; ***P < 0.001 vs. same-sex condition, indicated by color of asterisk(s), within the same picomole amount. Number of animals is indicated in respective legends: cage control (CC), exercise control (EC), food restricted control (FC), activity-based anorexia (ABA).

Significant interactions between sex × condition and condition × pmol of DA were observed (see Supplemental Table S8 for statistics). For comparisons across each treatment condition, a repeated-measures ANOVA was performed within each sex. In adolescent females, a significant main effect of condition was detected [F(3,185) = 2.97, *P* < 0.05]. Specifically, at 40 and 50 pmol of DA, female EC and FC rats had slower DA clearance relative to same-sex CCs (Fig. 5A). Clearance rates in female ABA rats were also greater at 40 and 50 pmol of DA relative to FC rats. Adolescent males likewise exhibited a significant main effect of condition [F(3,155) = 5.86, *P* < 0.01], but in stark contrast to females, male ABA rats exhibited significantly impaired DA clearance relative to CC at DA levels of 20 pmol and higher (Fig. 5B). As with females, male EC and FC rats exhibited significantly impaired DA clearance at 20, 40, and 50 pmol, compared with male CCs.

#### Adults.

Adult rats exhibited no significant interactions across sex, condition, and/or picomoles of DA (Supplemental Table S8; all *P* > 0.57), though there was an expected significant main effect of picomoles of DA [F(5,235) = 143, *P* < 0.001] (Fig. 5, C and D).

### Adolescent DAT and Serotonin Transporter Expression

**Fig. 6.**
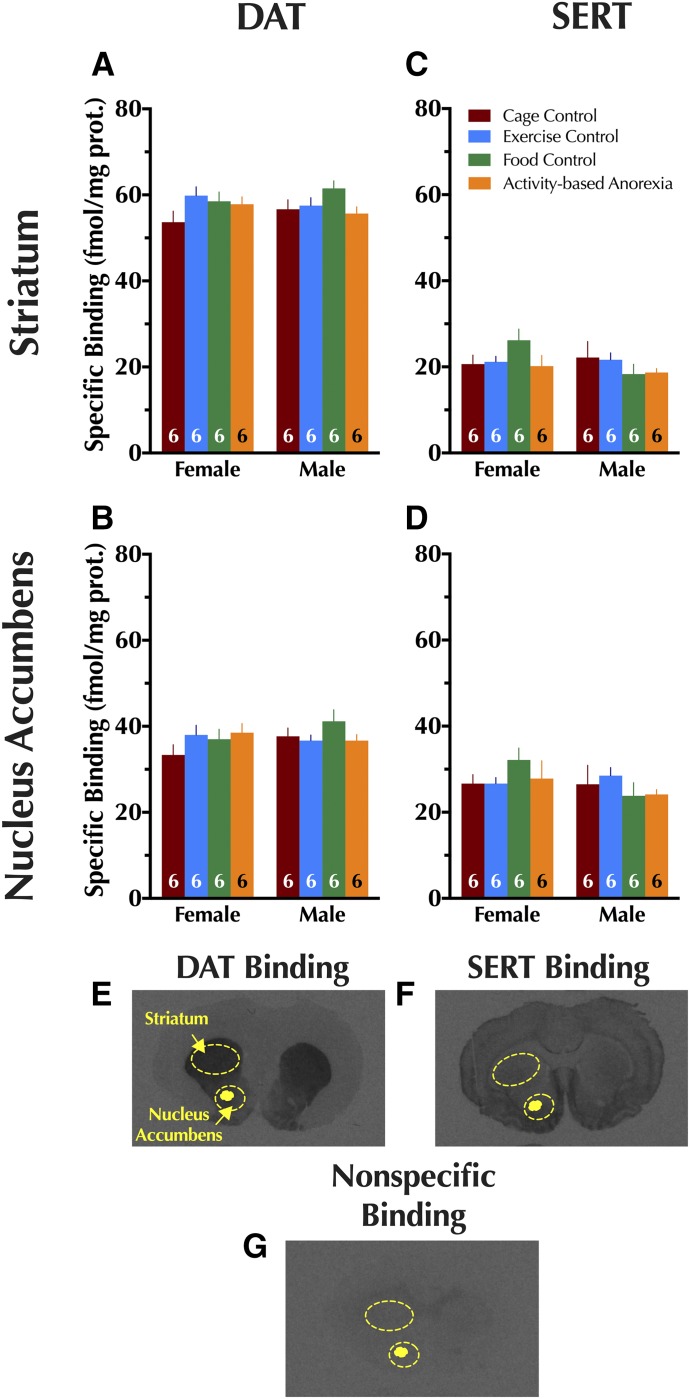
Quantitative autoradiography in adolescents for dopamine and serotonin transporter expression. Specific binding for dopamine transporter is shown in adolescent (A) striatum and (B) nucleus accumbens. Specific binding for serotonin transporter is also shown in adolescent (C) striatum and (D) nucleus accumbens. Representative brain sections illustrating (E) DAT, (F) SERT, and (G) nonspecific binding are shown with the analyzed regions for striatum and nucleus accumbens illustrated with dashed circles. Data were analyzed within each age group with a two-way (sex × condition) ANOVA. Data are graphed as mean + S.E.M. Number of animals is indicated at base of each bar.

Given that only adolescent rats exhibited functional changes in DAT as a result of condition, expression of DAT and serotonin transporter (SERT) was quantified using autoradiography in striatum and nucleus accumbens (NAc) of only adolescent animals. No significant differences in DAT or SERT expression were observed in either brain region in either sex, and no significant interactions between sex × condition were found (Fig. 6).

## Discussion

Our key findings indicate that during adolescence, the dopaminergic system possesses a functional plasticity impacted not only by sex but also by dietary and behavioral patterns, such as food restriction and exercise, in a manner not confounded by homeostatic body fluid disruptions or transporter expression changes. This responsive plasticity may feed forward to neurobehaviorally accelerate engagement in additional or continued unhealthy activities or compulsions, reshaping dopaminergic circuitry such that vulnerability to pervasive eating disorders or substance use disorders is perpetually augmented.

Being the first, to our knowledge, to apply the established adult ABA paradigm to *early* adolescence (P30), we observed striking vulnerability in adolescents compared with adults. Adult rats require at least 7 days to exhibit ≥20% starting body weight loss under these conditions (Routtenberg and Kuznesof, 1967); indeed, we observed minimal to no differences among treatment conditions in most adult endpoints. In contrast, adolescents responded so robustly that our experimentally planned 5-day paradigm was shortened to 4 days to facilitate survival for endpoint measures. This susceptibility of *early* adolescent rats parallels emergence in humans of pathologic behaviors and symptoms relating to eating disorders in early adolescence (age 13 years) (Micali et al., 2014; Nagl et al., 2016). The few studies using adolescent rats (all > P35) in an ABA paradigm have not validated the model (i.e., confirmed that key AN metabolic and hormonal manifestations are present), and seldom included all relevant controls, limiting interpretability of data (Kinzig and Hargrave, 2010; Aoki et al., 2012; Barbarich-Marsteller et al., 2013a,b; Chowdhury et al., 2014; Frintrop et al., 2018a,b). Moreover, the rapidity with which adolescent rats succumb to the adult-based ABA paradigm highlights the necessity for age-appropriate modifications that expand the experimental window beyond 4 days. Such a truncated window precludes the ability to investigate neurophysiological changes resulting from chronic ABA, and likewise hinders exploration of novel pharmacological interventions. Further, studies in adult rodents fail to reflect neural mechanisms active in the most vulnerable population, adolescents, during a time when brain maturation occurs. Considering AN’s high mortality (Arcelus et al., 2011; Preti et al., 2011; Keshaviah et al., 2014), as well as its long-term stability in those who survive (Nagl et al., 2016), a chronic ABA paradigm commencing during the vulnerable early adolescent period could provide a much-needed model in AN pathophysiology and treatment studies.

Blood hormone measurements further evidence the need for an optimized early adolescent ABA model. AN is characterized by substantial baseline reductions in circulating insulin and leptin, along with increases in ghrelin and cortisol (Monteleone et al., 2000; Nakahara et al., 2008; Karczewska-Kupczewska et al., 2010; Korek et al., 2013). With the exception of reduced leptin levels, none of these hormonal disruptions were mirrored in adolescents, underscoring the poor face validity of the current adult-based paradigm in adolescents. Recent efforts have prolonged survival of adolescent female rats in a modified ABA protocol (Frintrop et al., 2018b), but it remains to be seen whether this procedure elicits any sex-dependent effects, or reflects any hallmark blood hormone disruptions observed in AN patients. Another alternative approach to modeling AN uses a behavioral economics framework (Rowland et al., 2018), though how well this can be adapted to adolescents is undetermined.

Homeostatic body fluid maintenance, as evidenced by plasma osmolality and brain water weight, was not disrupted in FC and ABA adolescents, despite dramatic reductions in food and water consumption. Importantly, this suggests that the observed plasticity in DAT function is not confounded by dehydration, differences in DA diffusion, or changes in tortuosity at the carbon fiber microelectrode site.

The elevated hematocrit observed in adolescent ABA rats of both sexes, and adolescent male FC rats, is at odds with reports of reduced hematocrit levels in food restricted rats (Wojciak, 2014) and patients with severe AN [but see Symreng et al. (1985), Sabel et al. (2013)]. Instead, increased hematocrit is usually observed after excessive exercise in humans (Skarpańska-Stejnborn et al., 2015) but not rodents (Tian et al., 2012; Tang et al., 2019). Plasma protein levels were diminished only in adolescent female ABA rats, which could be attributable to muscle wasting or liver inflammation, both commonly observed in patients with AN (Rosen et al., 2017).

Despite the limitations of applying the adult ABA paradigm to adolescents, the current investigation revealed striking age- and sex-specific effects of exercise access plus food restriction on the locomotor-stimulating effects of the drug of abuse, cocaine. Cocaine-mediated blockade of DA uptake through DAT corresponds to cocaine-induced locomotor responses in Sprague-Dawley rats (Sabeti et al., 2002; Gulley et al., 2003). Thus, this behavior assay can serve as a proxy, at least in part, to indicate individual differences in DAT function as revealed by cocaine blockade.

To our knowledge, the influence of food restriction alone on adolescent or female locomotor activity in response to cocaine had not previously been explored, though the observed augmentation of cocaine response in adolescent males fits with previous, more extended periods of food restriction (1–2 weeks) in adult males (Bell et al., 1997). The absence of changes here in cocaine locomotor response after exercise alone across age and sex indicate that its effects take more than 4–5 days to emerge behaviorally, in agreement with multiple reports (Cosgrove et al., 2002; Smith et al., 2008a,b; Smith et al., 2011; Smith and Witte, 2012; Zlebnik et al., 2012). Most striking is that the combination of exercise plus food restriction in adolescent males substantially attenuated the locomotor response to cocaine, relative to FCs, but that this combination produced the largest behavioral response to cocaine in adolescent females. This is in accord with a disproportionately high prevalence of eating disorder diagnoses, particularly of AN, among females with comorbid substance use disorders [see Harrop and Marlatt (2010) for review]. The seemingly augmented response of ABA adolescent females to cocaine, versus the moderated response in ABA adolescent males, likely reflects underlying sex- and age-dependent plasticity in DAT function in response to the combination of food restriction and exercise.

Indeed, we directly investigated DAT function in dorsal striatum, where DAT expression is high, and DA signaling influences reward, activity, and eating behaviors (Sotak et al., 2005; Palmiter, 2007, 2008; Tomasi and Volkow, 2013). To our knowledge, this is the first characterization of in vivo DAT function in adolescent females, and the first evaluation of DAT functional changes after an ABA paradigm. We found a robust age difference in DA uptake across sexes, with faster clearance in adolescents compared with adults. Moreover, only in adolescents did ABA reveal a sex-specific effect, with dramatic functional DAT reductions observed in males but not females. Reductions in adolescent DAT function in ECs and FCs support the observed slowing of DA clearance in adolescent male ABAs, making the lack of attenuated DAT function in adolescent ABA females remarkable. Under prolonged (>4 days) ABA conditions, DAT function in adolescent females might eventually either increase, or persist at an adolescent CC-like level, reflecting clinical reports of significant DAT upregulation in female patients suffering from AN for an average of 10 years (Frieling et al., 2010). Optimization of the ABA paradigm for adolescents, to enable prolonged study with more of the characteristic hormonal disruptions in place, will permit study of this possibility. Importantly, the functional changes in DAT are not mirrored by any significant changes in total striatal DAT or SERT expression, the latter of which can transport DA under conditions of impaired DAT function (Larsen et al., 2011). These results suggest that DA clearance rate shifts are probably the result of changes in intrinsic activity and/or plasma membrane expression of DAT.

The sex-specific adolescent plasticity in dorsal striatum DAT function does not directly map on to cocaine-induced locomotor activity. This could be attributable to brain region–specific effects of cocaine on DA clearance, as cocaine-mediated blockade of DAT in NAc corresponds more closely to drug-induced changes in locomotor activity, compared to dorsal striatum (Sabeti et al., 2002). However, this is not attributable to any detectable changes in DAT expression or cocaine affinity in NAc (Gulley et al., 2003); indeed, we observed no significant differences in NAc DAT expression here. Thus, different cocaine-induced locomotor responses across conditions in female and male adolescents might reflect functional NAc DAT differences, whereas the functional changes measured using chronoamperometry in dorsal striatum could indicate DA clearance shifts that impact more habitual behaviors (e.g., home cage or running wheel activity).

Certainly, this plasticity could instead be secondary to other neurophysiological disruptions in the dopaminergic system (e.g., changes in DA release) or other neurotransmitter systems (Hillebrand et al., 2005; Atchley and Eckel, 2006), and could be influenced by apparent circadian changes in the adolescent ABA groups during the final 2 days of the paradigm (see Fig. 3). Nonetheless, the present findings suggest that striatal DAT function should be a focus of future studies into the mechanisms underlying eating disorder and drug abuse vulnerability. Moreover, a chronic ABA paradigm commencing during the vulnerable early adolescent period would provide a much-needed model to study AN pathophysiology and treatment.

## Abbreviations

ABA: activity-based anorexia
AN: anorexia nervosa
ANOVA: analysis of variance
AUC: area under the curve
CC: cage control
DA: dopamine
DAT: dopamine transporter
EC: exercise control
FC: food control
NAc: nucleus accumbens
RTI-55: methyl (1*R*,2*S*,3*S*)-3-(4-iodophenyl)-8-methyl-8-azabicyclo[3.2.1]octane-2-carboxylate
SERT: serotonin transporter
